# Implementation of a combined bioinformatics and experimental approach to address lncRNA mechanism of action: The example of NRIR

**DOI:** 10.3389/fmolb.2022.873847

**Published:** 2022-11-04

**Authors:** Barbara Mariotti, Costanza Di Blas, Flavia Bazzoni

**Affiliations:** Department of Medicine, Division of General Pathology, University of Verona, Verona, Italy

**Keywords:** NRIR, ISG, STAT1, STAT2, lncRNA structure, monocytes

## Abstract

In this study, we demonstrate the benefit of applying combined strategies to analyze lncRNA action based on bioinformatics and experimental information. This strategy was developed to identify the molecular function of negative regulator of interferon response (NRIR), a type I interferon-stimulated gene (ISG), that we have previously demonstrated to be involved in the upregulation of a subset of ISGs in LPS-stimulated human monocytes. In this study, we provide experimental evidence that NRIR is localized in cellular nuclei, enriched on the chromatin fraction, and upregulates ISGs acting at the transcriptional level. *In silico* analysis of secondary structures identified distinct NRIR structural domains, comprising putative DNA- and protein-binding regions. In parallel, the presence of a putative DNA-binding domain in NRIR and the five putative NRIR-binding sites in the promoter of NRIR-target genes support the function of NRIR as a transcriptional regulator of its target genes. By use of integrated experimental/bioinformatics approaches, comprising database and literature mining together with *in silico* analysis of putative NRIR-binding proteins, we identified a list of eight transcription factors (TFs) shared by the majority of NRIR-target genes and simultaneously able to bind TF binding sites enriched in the NRIR-target gene promoters. Among these TFs, the predicted NRIR:STAT interactions were experimentally validated by RIP assay.

## 1 Introduction

Long noncoding RNAs (lncRNAs), defined as transcripts >200 nucleotides in length and lacking protein-coding potential, represent the largest group of non-coding RNAs. In the most recent GENCODE V40 release, 18,805 annotated lncRNAs have been found in the human genome (Frankish et al., 2019). However, less than ∼3% of annotated lncRNAs have ascribed functions, and the mechanisms of action have been characterized for very few of them. Understanding how lncRNAs regulate cell functions represents the major challenge in the lncRNA field, mostly due to the fact that lncRNAs have an active role in controlling multiple regulatory layers, including chromosome architecture, chromatin modulation and epigenetic modification, transcription, RNA maturation, splicing, and translation (Dykes and Emanueli, 2017). Additionally, on average, lncRNAs display lower sequence conservation across species in comparison to proteins, thus complicating functional predictions based on primary sequence similarity (Derrien et al., 2012). Nevertheless, it has recently become clear that lncRNAs can fold into modular secondary or tertiary structures composed of multiple and heterogeneous domains capable of interacting with DNA, RNA, miRNA, and/or proteins, therefore influencing the lncRNA biological function (Rinn et al., 2020). There is no universal strategy for the characterization of the function of lncRNAs; nevertheless, several experimental and bioinformatics approaches have been developed to analyze lncRNA modes of action: characterization and/or prediction of lncRNA structure; identification of lncRNA subcellular localization; identification and/or prediction of lncRNA-interacting proteins. Each of these approaches by itself is informative but can provide only partial mechanistic information. In this study, we applied a combined strategy for intersecting structure prediction, bioinformatics, and experimental approaches, comprising database and literature mining together with *in silico* and *in vitro* analysis, to address the mechanism(s), whereby a given lncRNA exerts its function. This strategy was developed for gaining insights about how NRIR regulates the expression of selected IFN-stimulated genes (ISGs) in LPS-stimulated monocytes. In fact, we recently identified NRIR as an lncRNA upregulated in LPS-stimulated monocytes in a type I IFN-dependent manner and demonstrated that NRIR acts as a positive regulator of the expression of a subset of IFN-stimulated genes (ISGs) (Mariotti et al., 2019). Remarkably, the role of NRIR in the type I IFN pathway has been confirmed by demonstrations of an upregulated expression of this lncRNA in several diseases characterized by activation of the IFN response, such as systemic sclerosis (SSc) (Mariotti et al., 2019; Servaas et al., 2021), primary Sjögren’s syndrome (Peng et al., 2020), and systemic lupus erythematosus (Cao et al., 2019). In contrast, NRIR has been shown to play an important role in the pathogenesis of viral infections, where it contributes to viral replication by acting as a negative rather than a positive regulator of the expression of ISGs in hepatocytes or epithelial cells (Kambara et al., 2014; Wróblewska et al., 2017; Xu-Yang et al., 2017; Bayyurt et al., 2021). Such behavior is not uncommon among lncRNAs, and there are examples of lncRNAs that function as positive or as negative regulators of gene expression in a highly cell-type and/or stimulus-specific manner. On these bases, and since the present study draws on our precedent observations (Mariotti et al., 2019), the mechanism whereby NRIR modulates the induction of a set of ISGs was investigated in LPS-stimulated monocytes.

## 2 Materials and methods

### 2.1 Human monocyte purification, transfection, and culture

Human CD14^+^ monocytes were purified from buffy coats of healthy donors using the anti-CD14 microbeads (Miltenyi Biotec), on the autoMACs Pro Separator (Miltenyi Biotec) as described in Supplementary Materials. In selected experiments 8 × 10^6^ cells were transfected with 200 pmol NRIR-specific Silencer Select siRNA (Mariotti et al., 2019) or Silencer Select negative control #2 (Ambion, Thermo Scientific), using the Human Monocyte Nucleofector Kit and the AMAXA Nucleofector II (Lonza), according to the manufacturer’s protocol.

### 2.2 Cell fractionation

LPS-stimulated monocytes were resuspended for 10 min on ice in RLN1 buffer (50 mM Tris-HCl pH 8, 140 mM NaCl, 1.5 mM MgCl_2_, 0.5% NP-40) supplemented with 1 U/μl RNase Out (Invitrogen). After centrifugation at 300 g for 2 min, the supernatant was collected as the cytoplasmic fraction. The pellet was resuspended for 10 min on ice in RLN2 buffer (50 mM Tris-HCl, pH 8, 500 mM NaCl, 1.5 mM MgCl_2_, and 0.5% NP-40) supplemented with 1 U/μl RNase Out. Chromatin was pelleted at 16,100 g for 3 min. The supernatant represented the nuclear fraction. All fractions were resuspended in RLT buffer, and RNA was purified as described below.

### 2.3 RNA purification and gene expression analysis by RT-qPCR

Total RNA was purified with the RNeasy Mini Kit (Qiagen). Gene expression was analyzed as reported in Supplementary Material. The primers used in this study are listed in Supplementary Table S1. Expression data were reported as MNE (Muller et al., 2002) after normalization over ACTIN B.

### 2.4 *In silico* identification and characterization of putative NRIR-binding proteins

Identification of putative NRIR-binding proteins was performed using catRAPID Omics (parameters used: *Homo sapiens* library, RNA- and DNA-binding protein, full-length proteins (Livi et al., 2016), and the lncPRO algorithm (Lu et al., 2013) to identify interaction with the entire human proteome as reported in UniProt (https://www.uniprot.org/). Putative NRIR-binding proteins predicted by both algorithms were scored according to the lncPRO score, and the average calculated from the interaction score and the discriminative power obtained from catRAPID. Proteins common to lncPRO and catRAPID with a score higher than the average values were considered most probable NRIR-binding proteins. PANTHER protein families associated with the most probable NRIR-binding proteins were identified using the Gene List Analysis tool available at http://pantherdb.org/(Mi et al., 2013, 2019).

### 2.5 *In silico* identification and characterization of DNA binding elements of NRIR

The interaction between NRIR and DNA was analyzed with LongTarget (He et al., 2015). DNA sequences of the promoter region (5 Kb upstream of the transcriptional start site) of CXCL10, CXCL11, DDX58, ESPTI1, IFI44, IFIT2, and MX1 were recovered from Ensembl (https://www.ensembl.org/). The interaction between NRIR and each promoter was analyzed with LongTarget using default parameters. Identification of enriched motifs either in Triplex Forming Oligonucleotide (TFO) or Triplex Target Site (TTS) was performed using the Multiple Em for Motif Enrichment (MEME) tool (Bailey and Elkan, 1994). Only motifs enriched with an E-value<0.05 were further investigated. The similarity between identified motifs was evaluated with the TomTom motif comparison tool with default parameters (Gupta et al., 2007). The presence of putative NRIR-binding sequences in the promoters of CXCL10, CXCL11, DDX58, ESPTI1, IFI44, IFIT2, and MX1 was assessed by using the Motif Alignment and Search Tool (MAST) from the MEME suite (Bailey et al., 2015). Enrichment analysis of putative NRIR-binding motifs in the promoters of NRIR-target genes was performed using the Simple Enrichment Analysis (SEA) tool available in the MEME suite (Bailey et al., 2015). Promoter regions of the genes not affected by the NRIR target (Mariotti et al., 2019) were used as background.

### 2.6 NRIR structure analysis

NRIR structure was predicted using the RNAfold tool of the ViennaRNA suite (Gruber et al., 2008) with the following parameters: p -d2 –noLP. The results of the minimum free energy-based prediction were used in this study. For selected proteins, the putative binding region in the NRIR sequence was identified using CatRAPID Fragment with default parameters (Livi et al., 2016).

### 2.7 Identification of transcription factors involved in NRIR-target transcription

Transcription factors (TFs) involved in the transcription of NRIR-target genes were obtained from the ENCODE project database (Dunham et al., 2012; Davis et al., 2018), as well as from published data. In order to identify the TFs involved in the transcription of NRIR-target genes in an unsupervised manner, a score was assigned to each TF according to the following rules:

• No information about the TF for the specific promoter: score 0
• Evidence from the literature of a predicted putative transcription factor binding site (TFBS) in the specific promoter: score 2
• Experimental evidence from the literature of the TF recruitment to the specific promoter: score 6
• Evidence from ENCODE of the TF recruitment to the specific promoter: score 8
• TF recruitment to the specific promoter is reported both in literature and ENCODE: score 10

The matrix obtained by applying these scoring rules was subjected to hierarchical clustering using the *hclust* function in R, with *column_split* = 4.

### 2.8 Transcription factor binding site enrichment analysis

Identification of TFBSs enriched in NRIR-target genes was performed using PSCAN (Zambelli et al., 2009). The TF motifs available in Transfac were used in the analysis, and the promoters of the 47 genes unaffected by NRIR-silencing were used as background (Mariotti et al., 2019). For subsequent analysis, only TFBSs significantly (*p-value* < 0.05) enriched in the NRIR target were considered.

### 2.9 Nuclear RNA ImmunoPrecipitation

Nuclear RNA ImmunoPrecipitation (nRIP) was performed according to Zhao, (2015), with minor modifications. For each immunoprecipitation, 10^7^ CD14^+^ monocytes were treated with 100 ng/ml LPS for 4 h and harvested in ice-cold PBS prior to cell lysis. The protocol for cell lysis and nRIP analysis is detailed in Supplementary Material. Data are expressed as a percentage of the non-immunoprecipitated RNA (% of input) (Castellucci et al., 2015).

### 2.10 Statistical analysis

Data are expressed as mean ± SEM, unless otherwise indicated. Statistical evaluation was determined using two-way analysis of variance (ANOVA), followed by Bonferroni’s multiple comparisons test. *p*-value<0.05 was considered significant. GraphPad 8.0 was used.

Additional details are described in Supplementary Materials and Methods.

## 3 Results

### 3.1 Nuclear NRIR regulates the transcription of a subset of LPS-induced ISGs

**FIGURE 1:**
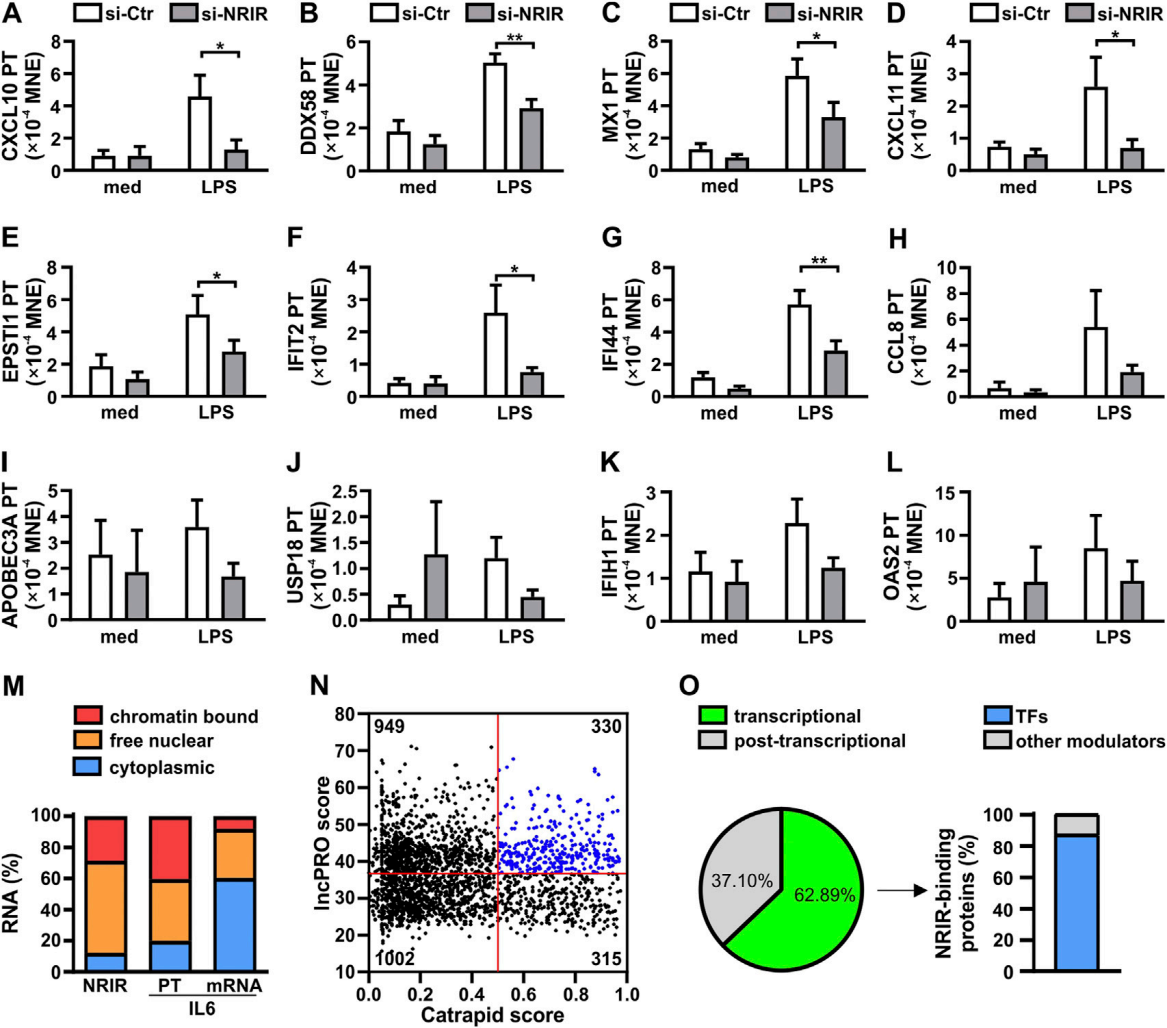
Nuclear NRIR regulates gene expression at the transcriptional level. **(A–L)** CD14^+^ monocytes were transfected with NRIR-specific siRNA (si-NRIR ) or control siRNA (si-CTR ) and stimulated with 100 ng/ml LPS for 4 h or left untreated (med). The expression of the primary transcript (PT) of CXCL10 **(A)**, DDX58 **(B)**, MX1 **(C)**, CXCL11 **(D)**, EPSTI1 **(E)**, IFIT2 **(F)**, IFI44 **(G)**, CCL8 **(H)**, APOBEC3A **(I)**, USP18 **(J)**, IFIH1 **(K)**, and OAS2 **(l)** was analyzed by RT-qPCR and expressed as MNE. Results are shown as mean ± SEM of 4–5 different experiments. *: *p* < 0.05, **: *p* < 0.01 according to two-way ANOVA followed by Bonferroni’s multiple comparisons test. **(M)** CD14^+^ monocytes were cultured for 8 h with LPS (100 ng/ml) and subjected to sub-cellular fractionation as described in Materials and Methods. Cytoplasmic, free nuclear, and chromatin-bound RNA were purified, and the expression of NRIR, IL-6 primary transcript (IL-6 PT), and IL-6 mRNA (IL-6) was analyzed by RT-qPCR. Data are expressed as percentages (%) of the total cell lysates. The mean of two independent experiments is shown. **(N)** Putative NRIR-binding proteins were identified using catRAPID and lncPRO as described in Material and Methods. The results of the analysis are shown as a scatter plot. Each dot represents a protein for which the catRAPID score (*x*-axis) and lncPRO score (*y*-axis) are shown. Red lines represent the threshold value for catRAPID score (score=0.5) and lncPRO score (score = 36.4). The number of proteins falling into the four subsets identified by the threshold is reported in each section. Blue dots highlight the most probable NRIR-binding proteins. **(O)** Panther protein family associated with the 330 proteins with the highest catRAPID and lncPRO score (N, blue dots). The percentage of NRIR-binding proteins associated with transcriptional (green) or post-transcriptional (grey) regulation of gene expression is represented as a pie chart. The percentage of transcription factors (TFs, blue) among the NRIR-binding proteins associated with transcriptional regulation is shown in the bar graph on the right.

Recently, we have demonstrated that NRIR plays a role in the induction of the expression of 15 out of 56 interferon-stimulated genes (ISGs) in human monocytes activated by LPS (Mariotti et al., 2019). In order to determine whether the upregulation of LPS-induced ISGs mediated by NRIR occurs at the transcriptional or post-transcriptional level, the expression of the primary transcript (PT) of the fifteen NRIR-target genes was analyzed by RT-qPCR using specific primer pairs in the same NRIR-silenced monocytes used in our previous work (Mariotti et al., 2019) (Supplementary Figure S1A). Under these conditions, the induction of 12 ISG transcriptions by LPS was impaired as compared to cells transfected with a scramble siRNA (Figure 1A-L). Specifically, a significant decrease of LPS-induced PTs for CXCL10 (-66.92%±11.05%, Figure 1A), DDX58 (-39.68%±9.88%, Figure 1B), MX1 (-41.96%±13.16%, Figure 1C), CXCL11 (-66.06%±9.04%, Figure 1D), EPSTI1 (-37.81%±11.47%, Figure 1E), IFIT2 (-57.10%±10.94%, Figure 1F), and IFI44 (-45.75%±11.59%, Figure 1G), was detected. Consistently, even though not statistically significant, reduction of CCL8 (-43.83%±15.74%, Figure 1H), APOBEC3A (-51.78%±12.51%, Figure 1I), USP18 (-60.86%±3.78%, Figure 1J), IFIH1 (-37.21%±12.54%, Figure 1K), and OAS2 (-50.49%±7.32%, Figure 1L) PTs was also observed 4 h after LPS stimulation in NRIR-silenced monocytes. Transcription of OAS3, IFITM3, and ISG15, previously shown as NRIR-target genes (Mariotti et al., 2019), could not be analyzed for technical reasons (see Supplementary Material and methods). Transcriptional upregulation of additional LPS-induced ISGs, namely, IRF7, IFIT1, and OASL, comprising the 41 protein-coding genes previously identified as induced by LPS in an NRIR-independent manner (Mariotti et al., 2019), was unaltered in NRIR-silenced monocytes (Supplementary Figure 1B–F), thus suggesting that NRIR is required for transcription of a subset of LPS-induced ISGs.

To support the role of NRIR in the upregulation of LPS-induced ISG transcription, localization of NRIR was investigated, by RT-qPCR, in cytoplasmic, nuclear, and chromatin-associated fractions obtained from LPS-stimulated human monocytes (Figure 1M). Data showed that NRIR is predominantly (87.64%±1.95%) localized in monocyte nuclei, with 32.72%±2.76% of it associated with chromatin. The purity of cellular fractions was demonstrated by the distribution of IL-6 mRNA and IL-6 PT, which were properly enriched in monocyte cytoplasm and nucleoplasm, respectively (Figure 1M).

LncRNAs can regulate transcription by a variety of mechanisms as they engage with proteins such as transcription factors, histone deacetylases, methyl-transferases, or chromatin remodeling complexes (Akkipeddi et al., 2020). Given the capability of NRIR to regulate transcription of its target genes, transcription factors (TFs) were among the expected NRIR-binding proteins. To support this hypothesis, *in silico* analysis of putative NRIR-binding proteins was performed by catRAPID and lncPRO. This analysis returned a list of 2,596 proteins predicted as putative NRIR-interacting proteins that were plotted as a function of their prediction scores (Figure 1N). The 330 proteins with a score higher than the average score for both the algorithms (Figure 1N, blue dots) were then subjected to functional classification using PANTHER (Supplementary Table S2). Remarkably, 63% of the proteins (n=139) were classified as involved in transcriptional regulation, and 37% (n=82) were functionally grouped as associated with post-transcriptional regulation (Figure 1O). Notably, 87% (n=121) of the proteins classified as a regulator of transcription specifically belong to the transcription factor class, thus strongly hinting to transcription factors as the most likely NRIR-interacting proteins (Figure 1O).

### 3.2 *In silico* prediction of NRIR structure

**FIGURE 2:**
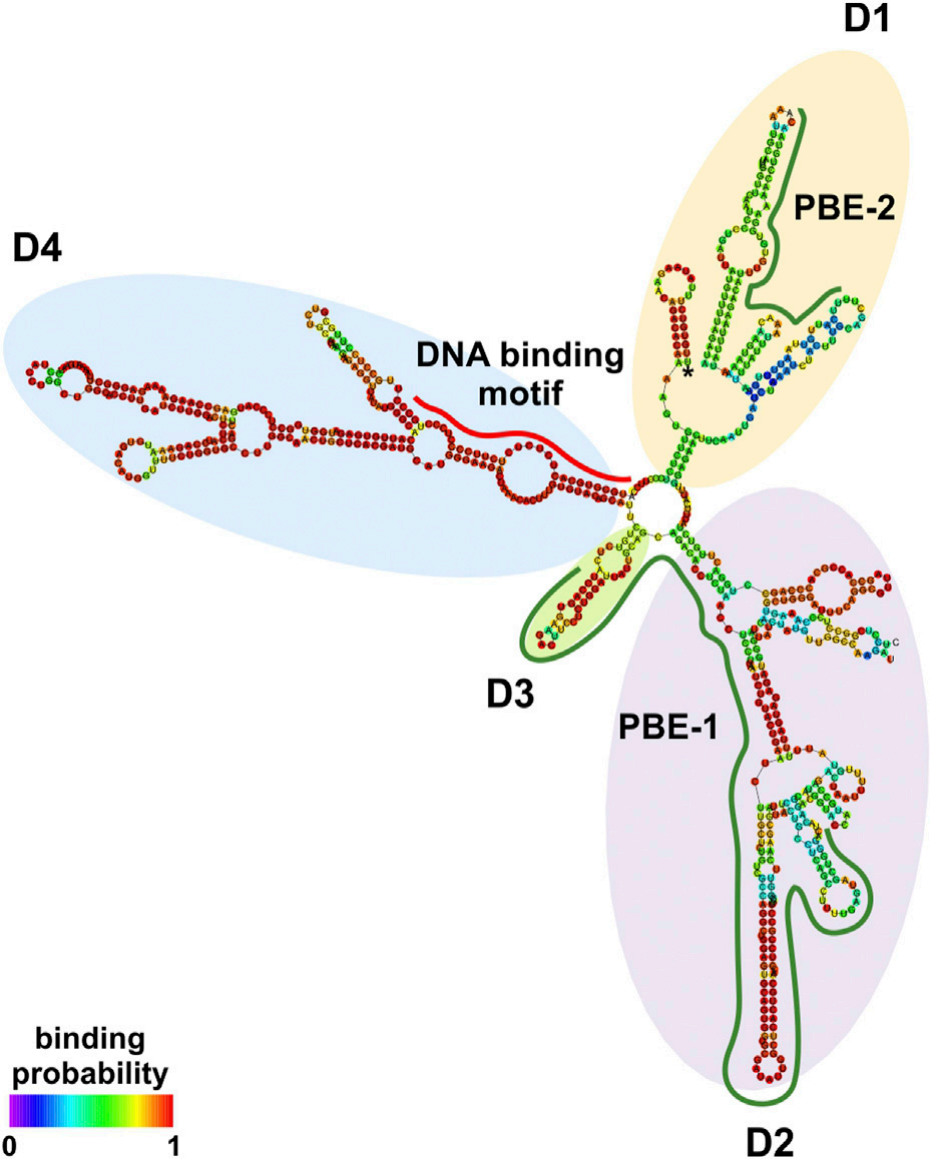
NRIR secondary structure. NRIR secondary structure was predicted by RNAfold as described in Material and Methods. CatRAPID fragment was used to identify protein-binding elements in the NRIR sequence. NRIR structure is colored according to nucleotide-binding probability obtained from RNAfold. Structural domains (D1 to D4) were highlighted with yellow (D1), purple (D2), green (D3), and light blue (D4) circles. A red line indicates the predicted DNA-binding motif, while green lines highlight the identified protein-binding elements (PBE-1 and PBE-2).

At the transcriptional level, a model is emerging whereby an lncRNA bridges DNA and protein by binding to chromatin and working as a scaffold for transcription factors and/or modifying protein complexes (Singh and Prasanth, 2013). LncRNA:protein and lncRNA:DNA interactions are mediated by interacting elements located in specific structural domains (Wang and Chang, 2011). NRIR secondary structure was investigated using RNAfold with the minimum free energy (MFE) and partition function. NRIR was predicted as a highly structured lncRNA composed of four main domains (D1 to D4) starting from a central four-way junction (Figure 2 and Supplementary Table S3). D1 and D2 appeared as two complex domains, composed of a total of three internal junctions: one five-way in D1, one five-way and one four-way junction in D2; nine terminal loops, 13 internal loops, and 13 helices (Figure 2). Differently, D3 and D4 were characterized by a lower degree of complexity than D1 and D2. D3 consisted of one helix, one external loop, and two internal loops (Figure 2). D4 was composed of two three-way internal junctions, three terminal loops, five internal loops, and five helices. Overall, the presence of highly complex domains (D1 and D2) and fewer complex domains (D3 and D4) suggested the co-existence of DNA-binding and protein-binding domains in NRIR.

### 3.3 Identification and characterization of putative DNA-binding domains in NRIR

**FIGURE 3:**
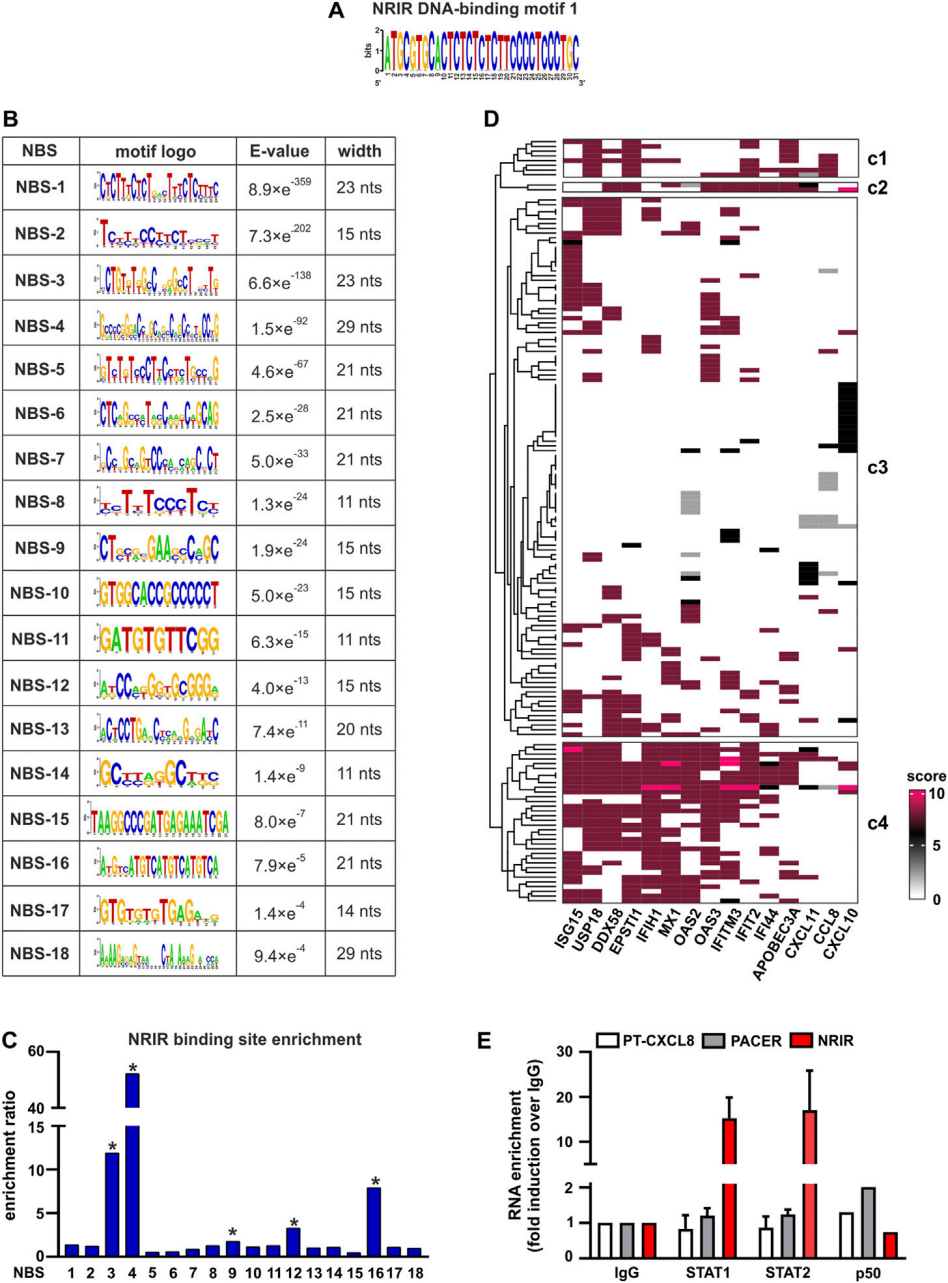
Identification of putative NRIR:DNA and NRIR: protein interactions. The interaction between NRIR and the promoters of its target genes (Figures 1A–G) was analyzed with LongTarget as described in Materials and Methods. **(A)** Putative DNA-binding motifs in the NRIR sequence were identified in the TFO1 using MEME. Motif-logo of the most significantly enriched NRIR DNA-binding motif is shown. **(B)** List of the 18 putative NRIR binding sequences (NBS) that were identified in the selected TTS using MEME (see Materials and Methods). The motif logo, enrichment E-value, and width of the motif (nts: nucleotides) are shown. **(C)** Relative enrichment analysis of the 18 NBS in the promoters of NRIR-target genes was performed using SEA. The enrichment ratio is shown. * *p*-value < 0.05 as determined by SEA analysis. **(D)** List of the transcription factors (TFs) involved in the transcription of NRIR-target genes was identified and scored as described in Material and Methods. The obtained scoring matrix was represented as a heatmap and subjected to hierarchical clustering analysis. Clusters were identified using the split argument of the Heatmap function in R. The obtained clusters are identified as c1 to c4. Names of the NRIR-target genes are shown under the heatmap. **(E)** NRIR, PACER, and CXCL8 primary transcript (PT-CXCL8) association with STAT1, STAT2, and p50 NF-κB was investigated with nuclear RIP. CD14^+^ monocytes were treated with LPS (100 ng/ml) for 4 h. Nuclear lysates were further incubated with 5 μg αSTAT1, αSTAT2, αp50 NF-κB, or control IgG antibodies. NRIR, PACER, and PT-CXCL8 expressions were analyzed by RT-qPCR. Data are shown as RNA enrichment expressed as fold induction over the IgG. Bars represent the mean standard deviation of three different experiments. One experiment out of two performed was shown for nRIP with αp50 NF-κB.

To fulfill its role as a transcriptional regulator, an lncRNA must contain a DNA-binding domain that mediates the formation of RNA:DNA triplex structures. The identification of putative DNA-binding motifs in NRIR and putative NRIR-binding sites in the promoters of the seven genes significantly modulated by NRIR (i.e. CXCL10, DDX58, MX1, CXCL11, EPSTI1, IFIT2, and IFI44, Figures 1A–G) was performed *in silico* by the use of LongTarget (He et al., 2015). LongTarget analysis returned a number of putative NRIR–DNA interactions that were grouped into triplex-forming oligonucleotides (TFO) identified in the NRIR sequence. TFO1, the best TFO of all the TFOs that were generated, was then identified. In parallel, LongTarget provided a list of triplex target sites (TTSs), complementary to the identified TFO, that represent the putative NRIR-binding sequences in the genomic regions analyzed (He et al., 2015). Analysis of promoter sequences of the selected NRIR-target genes led to identification of 220 NRIR: DNA interactions associated with TFO1 (Supplementary Table S4). All 220 sequences were subjected to a motif enrichment analysis by MEME in order to identify a shared motif. Three major motifs (herein called NRIR DNA-binding motifs, DBM) were identified (Supplementary Table S5), among which DBM-1 showed the best features in terms of enrichment (E-value=7.0×e^-3316^) and length (31 nts) (Figure 3A and Supplementary Table S5). Noticeably, DBM-2 and DBM-3 displayed a significant (*p*-value=8.98×e^-5^ and 6.71×e^-2^, respectively) homology with DBM-1 as evaluated by TomTom (Supplementary Table S5), suggesting that these two motifs may be part of the DBM-1 rather than distinct DBMs. Remarkably, and consistent with NRIR secondary structure, the DBM-1 is localized in the first helix of D4 (Figure 2) at position 45–75 nt (Supplementary Table S3), thus strengthening the likelihood that D4 represents the putative DNA-binding domain of NRIR.

The putative motifs recognized by DBM1 in the promoters of CXCL10, CXCL11, DDX58, ESPTI1, IFI44, IFIT2, and MX1 were identified by analyzing all the TTS complementary to TFO1. Motif enrichment analysis of TTS led to the identification of 18 significantly enriched motifs (E-value<0.05, Figure 3B), which from now on are called NRIR-binding sites (NBS). The presence of the 18 NBS in the promoter region of CXCL10, CXCL11, DDX58, ESPTI1, IFI44, IFIT2, and MX1 was assessed by *in silico* analysis using MAST. All the promoters contain at least 11 NBS. The number of sites for each NBS is shown in Supplementary Figure S2. In order to identify the NBS more likely involved in the capability of NRIR to specifically regulate the subset of LPS-induced ISGs, a relative motif enrichment analysis was performed using SEA. SEA identifies motifs that are relatively enriched in a set of input sequences, herein consisting of the promoter sequences of all the 15 NRIR-target genes, as compared to user-provided control sequences, herein represented by the promoter sequences of the 41 LPS-induced NRIR-independent genes (Mariotti et al., 2019). As a result, five NBS, namely NBS-3, NBS-4, NBS-9, NBS-12, and NBS-16, were significantly enriched in NRIR-target gene promoters (Figure 3C). Collectively, the identification of the DNA-binding domain 1 in NRIR and of five NRIR-binding sites in the promoter of NRIR-target genes strongly supported the role of NRIR as a transcriptional regulator of its target genes.

### 3.4 NRIR interacts with STAT1 and STAT2 in LPS-treated human monocytes

In order to identify TFs interacting with NRIR and concurrently involved in the regulation of NRIR-target ISGs, we first assessed the transcription factor-binding sites (TFBS) significantly over-represented in the promoters of the 15 NRIR-target genes. Fifty-nine TFBSs were found significantly enriched in NRIR-target gene promoters as compared to non-targeted gene promoters by PSCAN (Supplementary Table S6). In parallel, a search of the literature and of the ENCODE project database for proteins demonstrated to promote transcription of the 15 NRIR-target genes yielded a list of 158 TFs. Each of these 158 TFs was assigned a score, according to the criteria described in Material and methods. Hierarchical clustering analysis followed by a k-mean clustering uncovered a cluster of 34 TFs involved in the regulation of the majority of the NRIR-target genes (cluster 4, Figure 3D). The intersection of the list of 34 TFs with the list of the 59 TFBS enriched in NRIR-target genes returned eight TFs, namely, SP1, MYC, MAX, STAT1, TBP, STAT2, YY1, and IRF1 (Supplementary Figure S3), that were shared by the majority of NRIR-target genes and simultaneously were able to bind to the TFBS enriched in the NRIR-target gene promoters. Collectively, these data suggested a mechanism through which NRIR binding to the TFs selected according to the strategy described above promotes transcription of the selected NRIR-target genes.

The interaction profile of NRIR with each of the eight TFs identified was performed by catRAPID fragment. This analysis identified a principal protein binding element (PBE-1) spanning 300–480 nt (Supplementary Figure S4 and Supplementary Table S7), shared by all the TFs and overlapping the D2 and D3 domains in the NRIR structure (Figure 2). Interaction profiles of NRIR and MYC, STAT1, or SP1 detected a second protein binding element (PBE-2), spanning 660–730 nt (Supplementary Figure S4 and Supplementary Table S7), located in the D1 domain (Figure 2).

Among the eight TFs selected as described above, STAT1 and STAT2 have been previously demonstrated to be constitutively upregulated in monocytes from Systemic Sclerosis patients and to correlate with the expression of STAT-target genes (Van Der Kroef et al., 2019). In the same *in vivo* setting, NRIR has also been demonstrated to be constitutively upregulated, and to account for the type I IFN signature that is a hallmark of this pathology (Mariotti et al., 2019). Based on these experimental data, the existence of a causal link between STAT1/2 and NRIR was investigated by nuclear-RIP assay (nRIP). Freshly purified monocytes were treated with LPS for 4 h, and nuclear lysates were incubated with αSTAT1 or αSTAT2 antibodies or with IgG as a control. The presence of NRIR was assessed by RT-qPCR in αSTAT1, αSTAT2, and IgG immune precipitates (IPs). In parallel, in order to validate the nRIP assay, nuclear lysates were incubated with αp50 NF-κB antibodies, and the presence of PACER, a lncRNA already demonstrated to bind p50 NF-κB (Krawczyk and Emerson, 2014), was assessed by RT-qPCR. PACER was detected exclusively in αp50 NF-κB IPs, as it was undetectable in αSTAT1, αSTAT2, and IgG IPs. Remarkably, significant recruitment of NRIR was observed in both STAT1 and STAT2 IPs, but not in the control IgG IP nor in the αp50 NF-κB IP (Figure 3E), thus providing experimental validation of the predicted NRIR:STAT interactions.

## 4 Discussion

**FIGURE 4:**
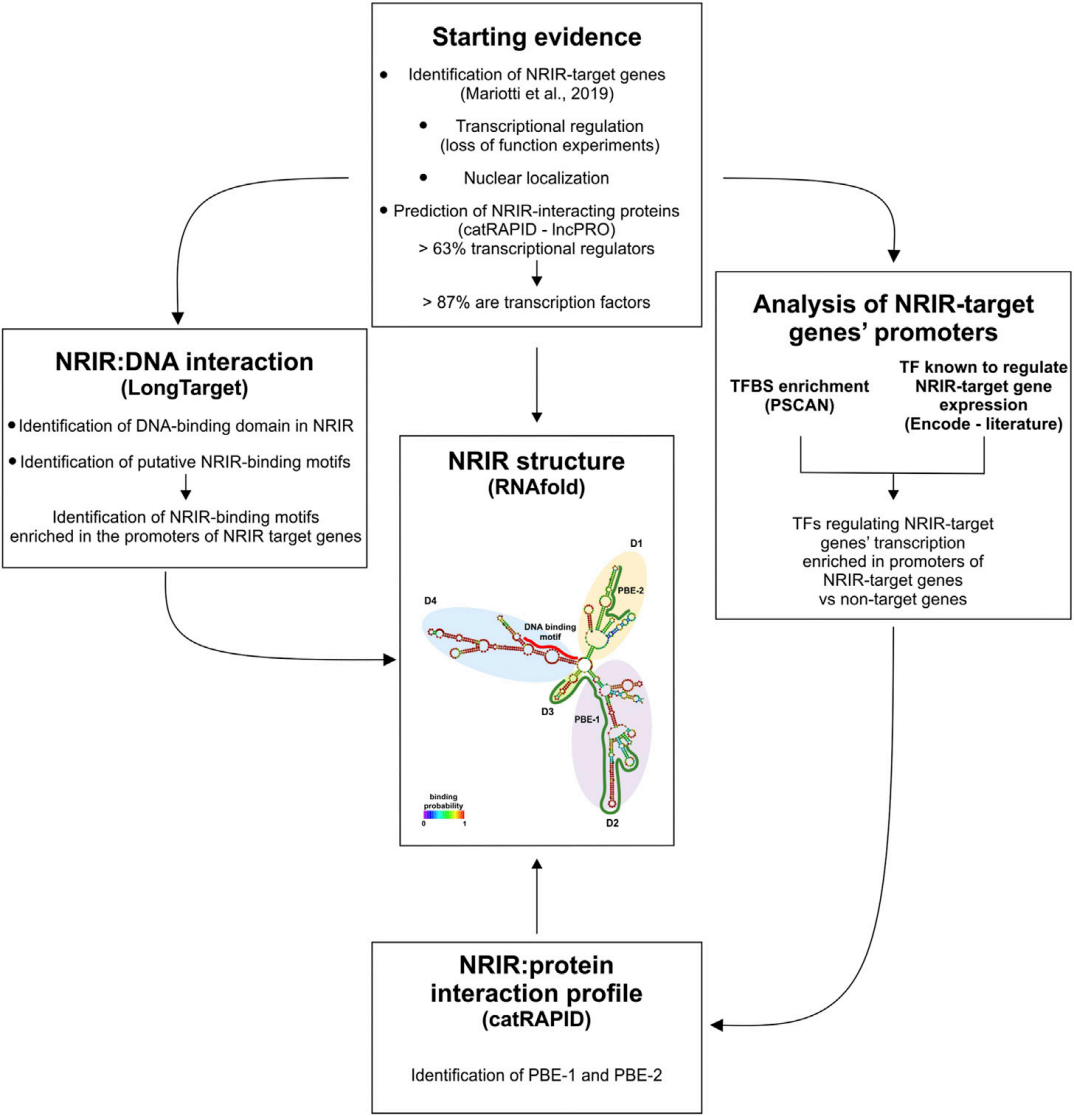
Schematic representation of the combined strategy used in this study. The combined strategy used in this study, together with the experimental and/or bioinformatic approach utilized, is depicted. PBE: protein-binding element; TF: transcription factor; TFBS: transcription factor-binding site.

In this work, a combined strategy, based on the integration of structure prediction, experimental data, and database mining, was implemented to investigate the NRIR mechanism of action (Figure 4). Each of these experimental approaches provided information about lncRNA function by itself. However, any single approach yielded partial information, and only the intersection of results from multiple strategies helped us identify the multiple functions of NRIR. At first, localization of NRIR coupled with evidence that upregulation of ISG expression occurs at the transcriptional level restricted the subsequent investigation to nuclear mechanisms involved in transcriptional regulation. Several studies point to an emerging model, whereby nuclear lncRNAs regulate transcription by bridging DNA and protein, binding to chromatin and serving as a scaffold for transcription factors and/or modifying protein complexes (Singh and Prasanth, 2013). Therefore, central to the identification of NRIR function was the characterization of its structure since the secondary structure of lncRNAs dictates their function. The RNAfold algorithm predicted a relatively complex secondary structure, consisting of four major domains (D1 to D4) departing from a central four-way junction. Structural properties of the NRIR domains meet the conditions required for an lncRNA to work as a transcriptional regulator, namely, that they must contain DNA- and protein-binding domains. In fact, the less complex structures of D3 and D4 were consistent with the presence of putative DNA-binding domains. Accordingly, the analysis of stable triple-helical NRIR-DNA structures by LongTarget led to identification of a precise NRIR DNA-binding motif (DBM-1), of 31 nt long. Localization of DBM-1 in the first helix of the D4 of NRIR reinforced the identification of D4 as the NRIR-DNA binding domain. At the level of the promoters of NRIR-dependent ISGs 18 motifs were identified as putative NRIR-binding sites (NBS). Remarkably, five of these NBS were significantly enriched in the promoters of NRIR-dependent ISGs, thus suggesting a molecular explanation discriminating NRIR-dependent from NRIR-independent ISGs. It should be pointed out that it is also possible that the NRIR-dependent ISGs, whose transcription level changes after NRIR knockdown, are the ones with low-affinity binding sites for NRIR. Conversely, genes with high-affinity NRIR binding sites may not be affected by the reduction of NRIR levels.

Mandatory partners of a nuclear lncRNA acting as a “guide” are lncRNA-interacting proteins that get tethered to the promoter/enhancer regions of the gene to be transcribed (Singh and Prasanth, 2013). Analysis of the other two NRIR domains, D1 and D2, identified tandem stem-loop structures that are predictive of putative protein-binding regions. In fact, the D1- and D2-stem-loop structures closely resemble the structures of roX2 (Ilik et al., 2013) and of Xist Repeat A (Zhao et al., 2008), previously reported to mediate lncRNA–protein interaction. Nuclear proteins known to interact with lncRNAs can belong to different classes of transcriptional regulators, including histone deacetylases, methyl-transferases or chromatin remodeling complexes, and transcription factors (Singh and Prasanth, 2013). Data from different approaches carried on in parallel converged in predicting transcription factors as the most probable NRIR-interacting proteins. In fact, 63% of the proteins commonly predicted by two independent algorithms (catRAPID and lncPRO) to be able to bind NRIR were functionally classified as transcriptional regulators. Remarkably, the vast majority of these transcriptional regulators were categorized as transcription factors. Transcriptional regulation by lncRNAs *via* recruitment of transcription factors to the promoters of target genes is not an uncommon mechanism and has been already described in the case, for instance, of PACER (Krawczyk and Emerson, 2014), RMST (Ng et al., 2012), lncSOX4 (Chen et al., 2016) and HOTAIR (Li et al., 2016). The intersection of the list of TF binding sites significantly enriched in the promoters of NRIR-dependent genes with the list of TFs common for the majority of the NRIR-dependent ISGs allowed us to restrict the list of potential NRIR-binding TFs. Utilizing this strategy, eight TFs simultaneously shared by NRIR-dependent ISGs and recognized by TF-binding sites enriched in the promoters of the same NRIR-dependent ISGs were identified. The soundness of this rationale was confirmed by the analysis of the interaction profile of NRIR with each of these eight TFs, which led to the identification of protein-binding elements located in the structural putative protein-binding domains D1 and D2, as predicted.

As proof of principle, a direct interaction between NRIR and STAT1/STAT2 was experimentally validated by nuclear-RIP assay. Collectively, *in silico* and *in vitro* data converged to demonstrate that NRIR upregulates transcription of a subset of ISGs by guiding STAT1 and STAT2 to the promoters of its target genes. This model is consistent with the emerging general mechanism, whereby at the transcriptional level, lncRNAs bridge DNA and proteins by binding to chromatin and working as a scaffold for transcription factors (Singh and Prasanth, 2013). STAT1 and STAT2 heterodimers associate with interferon regulatory factor 9 (IRF9) to form the transcriptionally active IFN-stimulated gene factor 3 (ISGF3) complex that controls gene expression by binding to interferon-stimulated response elements (ISRE) in ISG promoters (Levy and Darnell, 2002). Although the expression of a majority of type I IFN-induced genes is attributable to the activation of the canonical ISGF3, an increasing body of evidence showed that alternative STAT complexes, containing IRF9 and either STAT1 or STAT2, but not both, also form upon type I IFN stimulation in a cell-specific manner and have the potential to control ISG expression (Fink and Grandvaux, 2013; Majoros et al., 2017).

In this complex scenario, STAT1 and STAT2 are certainly able to modulate the transcription of more ISGs than the subset herein studied. Whether and how the transcriptional activity of different STAT1 and STAT2 complexes are modulated by NRIR needs to be determined. Moreover, since a growing number of lncRNAs have been demonstrated to interact with more than one protein partner, additional protein partners of NRIR cannot be excluded. Thus, the predicted interaction of NRIR with the other five TFs, namely SP1, MYC, MAX, TBP, YY1, and IRF1, should be experimentally tested. Finally, the identification of NRIR-interacting proteins in hepatocytes or epithelial cells, where NRIR has been demonstrated to act as a negative regulator of ISGs expression, might provide further insight into the mechanism, whereby NRIR acts either as a positive or a negative regulator of ISG gene transcription, according to the cellular context.

## Data Availability

The raw data supporting the conclusion of this article will be made available by the authors, without undue reservation.
