# Supplementary material for: Implementation of a combined bioinformatics and experimental approach to address lncRNA mechanism of action: The example of NRIR
Source: Front Mol Biosci. 2022 Nov 4;9:873847. doi: 10.3389/fmolb.2022.873847 (PMC9671926; doi:10.3389/fmolb.2022.873847)
Supplement: Supplementary file 1 [file DataSheet1.PDF]

Supplementary Material

Implementation of a combined bioinformatics and experimental approach to address lncRNA mechanism of action: the example of NRIR

Barbara Mariotti[1], Costanza Di Blas[1] and Flavia Bazzoni[1]

[1]Department of Medicine, Division of General Pathology, University of Verona, Verona, Italy

Correspondence:

Dr.ssa Barbara Mariotti,

Division of General Pathology

Department of Medicine

University of Verona

Strada Le Grazie 8, 37134

Tel: +39 045 802 7555

barbara.mariotti@univr.it

Supplementary Material

Supplementary Materials and methods

Human monocyte purification, transfection and culture

Human CD14+ monocytes were purified from buffy coats of healthy donors after centrifugation over Ficoll-Paque gradient. Briefly, CD14+ monocytes were purified from PBMCs using the anti-CD14 microbeads (Miltenyi Biotec), on the autoMACs Pro Separator (Miltenyi Biotec) according to manufacturer's protocol. Purity of monocyte preparations was usually >98%. Monocytes ($3x10^6$ cells/ml) were cultured in RPMI 1640 (Gibco) supplemented with 10% FCS (<0.5 EU/ml; Sigma-Aldrich) and 2 mM Glu in the presence or absence of 100 ng/ml ultra-pure lipopolysaccharide (LPS, from E. coli strain O111:B4, InvivoGen, San Diego, CA, USA) as indicated. In selected experiments $8x10^6$ were transfected with 200 pmol NRIR-specific Silencer Select siRNA (Mariotti et al., 2019) or Silencer Select negative control #2 (Ambion, Thermo Scientific), using the Human Monocyte Nucleofector Kit and the AMAXA Nucleofector II device (Lonza), according to the manufacturer's protocol. Once transfected, cells were plated in recovery medium [50% RPMI 1640 + 10% FCS + 2 mM Glu, and 50% IMDM (Lonza) + 10% FCS + 2 mM Glu], at $3x10^6$ cells/ml overnight. The next day, medium was changed to RPMI 1640 + 10% FCS + 2 mM Glu, and cells were stimulated as indicated.

RNA purification and gene expression analysis by RT-qPCR

Total RNA was purified with the RNeasy Mini Kit (Qiagen), according to the manufacturer's instructions. DNAse treatment on column was performed using the RNAse Free DNase I set, Qiagen. RNA quantification and purity were assessed at the Nanodrop 2000 spectrophotometer (Thermo Scientific). RNA samples were reverse transcribed using 5 ng/μl random primers, 1 U/μl RNase inhibitor (RNAse Out, Invitrogen) and 5 U/μl reverse transcriptase (SuperScript III, Invitrogen), according to manufacturer's instruction. NRIR expression was quantified by RT-qPCR from 9 ng RNA-equivalent cDNA in the presence of SYBR Select Master Mix (ThermoFisher Scientific, Applied Biosystems) and 400 nM specific primers (Supplementary Table S1), on the ViiA™ 7 Real-Time PCR System (ThermoFisher Scientific, Applied Biosystems) using the standard protocol. Expression of primary transcripts (PTs) and mRNAs was quantified by RT-qPCR from 9 ng RNA-equivalent cDNA in the presence of Fast SYBR Green Master mix (ThermoFisher Scientific, Applied Biosystems) and 200 nM of specific primer pairs (Supplementary Table S1), on the ViiA™ 7 Real-Time PCR System (ThermoFisher Scientific, Applied Biosystems). Primers were designed using OligoExplorer. Primers detecting primary transcripts (PTs) were designed from an intron region of the selected genes. For three out of the fifteen previously described NRIR target genes, it was not possible to design specific primer pairs or Ct value were too high in all the experimental condition to be further considered (Ct >32). Data were analyzed with Q-Gene software and gene expression was reported as mean normalized expression (MNE (Muller et al., 2002)) after normalization over the stably expressed ACTIN B.

Nuclear RNA ImmunoPrecipitation (nRIP)

Nuclear RNA ImmunoPrecipitation (nRIP) was performed according to Zao et al (Zhao, 2015) with minor modifications. For each immunoprecipitation, 10^7 CD14+ monocytes were treated with 100 ng/ml LPS for 4 h and harvested in ice-cold PBS prior to cell lysis. Cells were suspended in 100 μL PLB buffer [10 mM HEPES pH 7.0 + 100 mM KCl + 5 mM $MgCl_2$ + 0.5% NP40 + H_2O] supplemented with 1000 U/ml RNAseOUT (Thermo Fisher Scientific), 5 μM Vanidyl Ribonucleoside Complexes (VRC), 5 μg/mL leupeptin, 5 μg/mL pepstatin, 1 mM, phenylmethanesulphonylfluoride (PMSF), 1

mM Na_3VO_4, 20 μM phenylarsine oxide (PAO), and 50 mM NaF (PLB+i), for 5 minutes on ice and centrifuge 15 minutes at 2.500 g at 4°C. The nuclear pellet was further resuspended in 100 μL PLB+i and homogenized with a 30 G needle. DNA was digested with 400 U/ml of RNAse free DNAse (RNAse Free DNase I set, Qiagen) for 15 minutes at room temperature. 100 μL of the obtained lysate were suspended in 500 μl NT2 supplemented with 1000 U/ml RNAseOUT (Thermo Fisher Scientific), 5 μM VRC, 5 μg/mL leupeptin, 5 μg/mL pepstatin, 1 mM PMSF, 1 mM Na_3VO_4, 20 μM PAO, and 50 mM NaF and incubated over night at 4°C in the presence of 5 μg αSTAT1 (SC-346, Santa Cruz Biotechnology) or αSTAT2 (SC-476, Santa Cruz Biotechnology), αNFκB p50 (SC-7178, Santa Cruz Biotechnology) polyclonal antibodies or rabbit polyclonal IgG as control, followed by incubation with 20 μl Dynabeads Protein A (Thermo Fisher Scientific) for 4 h at 4°C. Beads were washed with NT2 buffer and then resuspended in 600 μl RLT supplemented with 0.143 mM β-mercaptoethanol. RNA was extracted and RT-qPCR was performed as previously described (Rossato et al., 2012). Data are expressed as percentage over the non-immunoprecipitated RNA (% of input, (Castellucci et al., 2015)).

Supplementary Material

Supplementary Table S1 Oligonucleotides sequences

RT-qPCR Primers		
	Sequence	
**RT-qPCR Target**	**Forward**	**Reverse**
**ACTIN B**	CATCGAGCACGGCATCGTCA	TAGCACAGCCTGGATAGCAAC
**IL6 mRNA**	CAAACAAATTCGGTACATCCTC	CAAGTCTCCTCATTGAATCCA
**NRIR**	CTGTCTCATCCAGTGAAGAC	TTGCAGTGAGCCAATATCGC
**APOBEC3A PT**	ACCTACCTGTGCTACGAAG	GAATGTCCCTGGATTGGAAG
**CCL8 PT**	TTGAGAACAACCCAGAAACC	CACTGTCTCCTCCCAAAAC
**CXCL8 PT**	ATTGAGAGTGGACCACACTG	ACTACTGTAATCCTAACACCTG
**CXCL10 PT**	GCTGATATGGGGATATAGGTTC	GCAGGTACAGCGTACAGTTC
**CXCL11 PT**	CCTTGGCTGTGATATTGTGTG	GTCCACCATTTCTGTGCTAAAG
**DDX58 PT**	CCTGGACCCTACCTACATC	CACCTCGCTGGAACTCAG
**EPSTI1 PT**	AACAGGTAAGGGTCAACAAG	GGATGAGAGGTACTATGGTC
**IFI44 PT**	AGGACTGCTGGCTTACTTGC	CGATGGGGAATCAATGTAGTC
**IFIH1 PT**	ATGGAGGAGGAACTGTTGAC	GGGCAAACGCACAAGGAAG
**IFIT1 PT**	TCTAGCCAGCATATGAAGCCAA	CAGACTATCCTTGACCTGATGA
**IFIT2 PT**	ATTTGCCATGCTCCCATTTC	TGCCCTTTGAGGTGCTTTAG
**IL6 PT**	ACATCCTCGACGGCATCTCAG	CCCAGCAAAGACCTCCTAATG
**IRF7 PT**	TGGCTCCTTGGAGAGATCAG	TGAAGAGGGGGACAGAACAC
**MX1 PT**	GAGAGGCAAGGTCAGTTAC	CCAAACAGGGGGTACTCAC
**OAS2 PT**	GGATGCCTGCCACTCAATG	CAACCACTTCGTGAACAGAC
**OASL PT**	GGACCGTGGAGGAGTTTCTG	GTGGGATGGGGTATTCTGAG
**PACER**	CGCCGTGTCTGGTCTGTAC	CAAGGAGGGGTGAAGGTAC
**USP18 PT**	GTCTCCCCAAACATTTATCTCC	CCAAACGCCTTGCTCATTCG

PT: primary transcript

Supplementary Figure S1

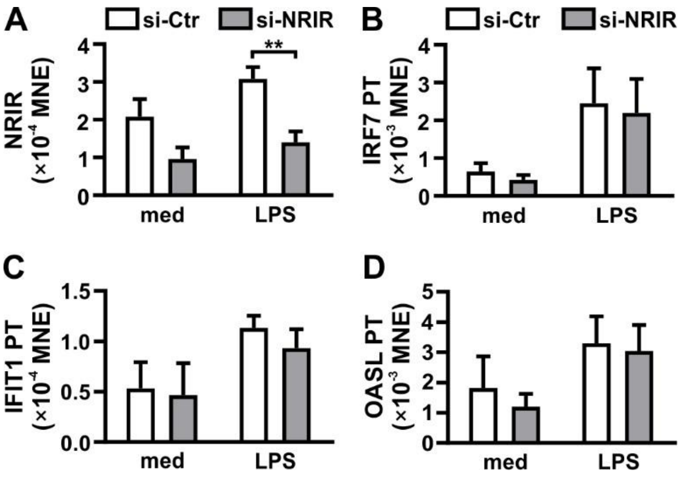

Supplementary Figure S1 Expression of IFIT1, IRF7 and OASL primary transcripts is not affected by NRIR silencing

CD14+ monocytes were transfected with NRIR specific siRNA (si-NRIR, grey bars) or control siRNA (si-Ctr, white bars) and stimulated with 100 ng/mL LPS for 4 hours or left untreated (med). The expression of NRIR (A) or of the primary transcript (PT) of IRF7 (B), IFIT1 (B) and OASL (D) was analyzed by RT-qPCR. Data are reported as MNE and shown the mean ± SEM of 4-5 different experiments. **: $p<0.01$ according to two-way ANOVA followed by Bonferroni's multiple comparisons test.

Supplementary Table S2 **Distribution of the NRIR-interacting proteins according to Panther protein classes**

Panther protein class name	Panther protein class ID	n
protein modifying enzyme	PC00260	8
scaffold/adaptor protein	PC00226	2
membrane traffic protein	PC00150	1
chaperone	PC00072	1
protein-binding activity modulator	PC00095	1
DNA metabolism protein	PC00009	9
general transcription factor	PC00259	6
DNA-directed RNA polymerase	PC00019	1
RNA metabolism protein	PC00031	46
transmembrane signal receptor	PC00197	1
calcium-binding protein	PC00060	1
viral or transposable element protein	PC00237	1
gene-specific transcriptional regulator	PC00264	115
translational protein	PC00263	13
metabolite interconversion enzyme	PC00262	9
chromatin/chromatin-binding, or -regulatory protein	PC00077	6

Terms were merged in the "transcriptional" (green), "post-transcriptional" (no colour) groups in Figure 1O.

Supplementary Table S3 **Position of structural domains and interacting elements in NRIR sequence**

Structural domain<br>Interacting element	Start<br>(nt position)	End<br>(nt position)
**Domain D1**	655	37
**Domain D2**	332	646
**Domain D3**	292	330
**Domain D4**	45	290
**DNA-binding motif 1**	45	75

Nt: nucleotide

Supplementary Table S4 Number of NRIR-promoter interactions identified in NRIR-target ISGs gene promoters.

Gene	number of interactions
**CXCL10**	66
**CXCL11**	66
**DDX58**	7
**EPSTI1**	27
**IFI44**	10
**IFIT2**	38
**MX1**	6
**Total number of interactions**	220

Supplementary Table S5 Motif enrichment analysis of TFO1

NRIR motif ID	sequence	E-value	length (nts)	match with DNA-binding motif 1 (p-value)
**DBM-1**	ATGCGTGCACTCTCTCTCTTCCCCTCCCTGC	$7.0 \times e^{-3316}$	31	
**DBM-2**	CTCTCCCTTCC	$1.3 \times e^{-723}$	11	$8.98 \times e^{-5}$
**DBM-3**	CCTTGCCTCCT	$7.0 \times e^{-420}$	11	$6.71\times e^{-2}$

DBM: DNA-binding motif; nts: nucleotides

Supplementary Material

Supplementary Figure S2

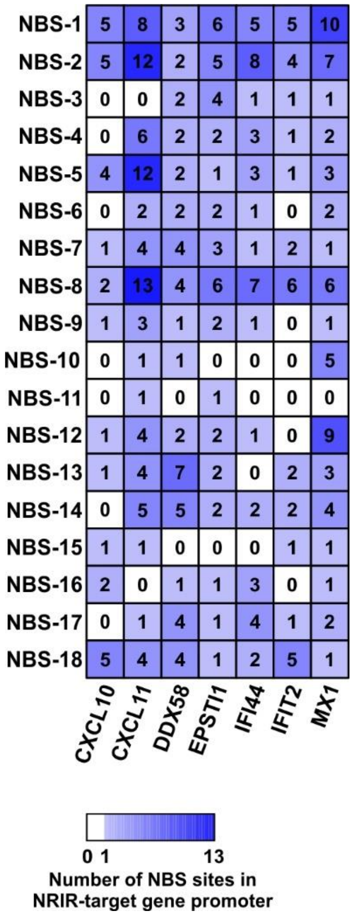

Supplementary Figure S2   Number of NBS sites in the promoter of NRIR-target genes.

The presence of the eighteen NRIR binding sequences (NBS) in the promoter of CXCL10, CXCL11, DDX58, EPSTI1 IFI44, IFIT2 and MX1 was determined using MAST (see Materials and Methods). Results are shown as heatmap, coloured according to the number of NBS sites found in each promoter (white: 0 site; light blue: 1 site; dark blue: 13 sites). The number of sites for each NBS in each promoter is also reported.

Supplementary Table S6 **Transcription factor binding site enrichment results.**

TF NAME	matrix ID	z-score	p-value	sample	background
**V$SOX9_B1**	M00410	4.14845	3.37E-06	0.913084	0.898309
**V$PBX1_01**	M00096	3.63538	6.27E-05	0.964481	0.952846
**V$GATA1_03**	M00127	3.58765	6.66E-05	0.932922	0.922661
**V$CEBP_Q2**	M00190	3.52629	7.86E-05	0.915062	0.904142
**V$IRF7_01**	M00453	2.94674	0.000396974	0.878707	0.867229
**V$NFAT_Q6**	M00302	2.91663	0.000784189	0.964588	0.957166
**V$CLOX_01**	M00103	2.92234	0.000841357	0.854262	0.839179
**V$CETS1P54_02**	M00074	2.8642	0.000860972	0.94216	0.936111
**V$CDP_02**	M00102	2.89203	0.000895703	0.859268	0.844609
**V$STAT_01**	M00223	2.69781	0.00117352	0.813953	0.804916
**V$HOX13_01**	M00023	2.79222	0.00133341	0.808122	0.801178
**V$MYCMAX_02**	M00123	2.75248	0.00143947	0.923154	0.913747
**V$STAT5B_01**	M00459	2.61309	0.00241043	0.879609	0.867159
**V$USF_C**	M00217	2.58735	0.00249559	0.963088	0.95527
**V$EVI1_04**	M00081	2.68541	0.00250218	0.925234	0.916582
**V$NMYC_01**	M00055	2.43193	0.00362839	0.90963	0.897742
**V$ZIC2_01**	M00449	2.42937	0.00412218	0.92471	0.913225
**V$TATA_01**	M00252	2.47219	0.00431743	0.91477	0.90643
**V$SREBP1_01**	M00220	2.34344	0.00508296	0.924977	0.912941
**V$CP2_01**	M00072	2.39173	0.00518825	0.944939	0.936168
**V$NCX_01**	M00484	2.27792	0.00540651	0.90134	0.893281
**V$OLF1_01**	M00261	2.26842	0.00553625	0.866328	0.859
**V$NFY_Q6**	M00185	2.29828	0.0062122	0.925748	0.918035
**V$YY1_02**	M00069	2.23454	0.00721248	0.83344	0.826092
**V$GATA_C**	M00203	2.34576	0.00763866	0.947919	0.940213
**V$NFKAPPAB50_01**	M00051	2.25852	0.00782566	0.893468	0.881007
**V$STAT1_03**	M00496	2.21936	0.00889472	0.899246	0.889038
**V$FREAC3_01**	M00291	2.04482	0.0106273	0.874175	0.864834
**V$STAT5A_04**	M00499	2.21342	0.0111605	0.997474	0.995742
**V$GATA6_01**	M00462	2.16832	0.0124838	0.969315	0.96404
**V$PAX4_03**	M00378	2.0763	0.0140998	0.955832	0.950793
**V$PAX5_01**	M00143	2.00355	0.014462	0.846496	0.841313
**V$AP4_Q5**	M00175	2.0286	0.0149407	0.965066	0.959804
**V$P53_02**	M00272	2.0139	0.0150925	0.956691	0.950621
**V$XFD1_01**	M00267	2.05954	0.0151588	0.930967	0.920947
**V$EN1_01**	M00396	1.9708	0.0159863	0.988465	0.985211
**V$ISRE_01**	M00258	1.92905	0.0167769	0.868403	0.85852
**V$GATA1_06**	M00347	1.95368	0.0177056	0.942263	0.932786
**V$GATA1_04**	M00128	1.95069	0.0192869	0.935607	0.928169
**V$HTF_01**	M00538	1.75943	0.0214629	0.820614	0.814859
**V$AP4_Q6**	M00176	1.88892	0.0220438	0.953285	0.94618
**V$AP4_01**	M00005	1.84665	0.0227211	0.870392	0.863666
**V$GATA1_02**	M00126	1.84418	0.0232175	0.91438	0.907225
**V$FOXJ2_01**	M00422	1.88791	0.0243191	0.943291	0.93469
**V$RFX1_01**	M00280	1.79118	0.0270595	0.867236	0.861367

**V$PAX4_02**	M00377	1.82983	0.02817	0.947917	0.942048
**V$CMYB_01**	M00004	1.75238	0.0298689	0.842786	0.836673
**V$FREAC7_01**	M00293	1.80328	0.0322023	0.921008	0.912024
**V$CHOP_01**	M00249	1.67739	0.0334983	0.889243	0.883691
**V$PAX5_02**	M00144	1.72442	0.0336242	0.819314	0.815695
**V$CDXA_01**	M00100	1.75097	0.0338864	0.989846	0.986127
**V$MRF2_01**	M00454	1.66267	0.0363572	0.893799	0.889096
**V$SP1_01**	M00008	1.68525	0.0368292	0.95327	0.948442
**V$GATA3_02**	M00350	1.68203	0.0392651	0.933187	0.925764
**V$MZF1_01**	M00083	1.61699	0.0403224	0.983942	0.979886
**V$MEF2_03**	M00232	1.60528	0.0432606	0.855418	0.84973
**V$CDXA_02**	M00101	1.69104	0.0438063	0.999474	0.998523
**V$CREB_01**	M00039	1.58035	0.0460401	0.913134	0.907135
**V$TCF11_01**	M00285	1.55767	0.0482382	0.963302	0.959523

TF: transcription factor

Supplementary Figure S3

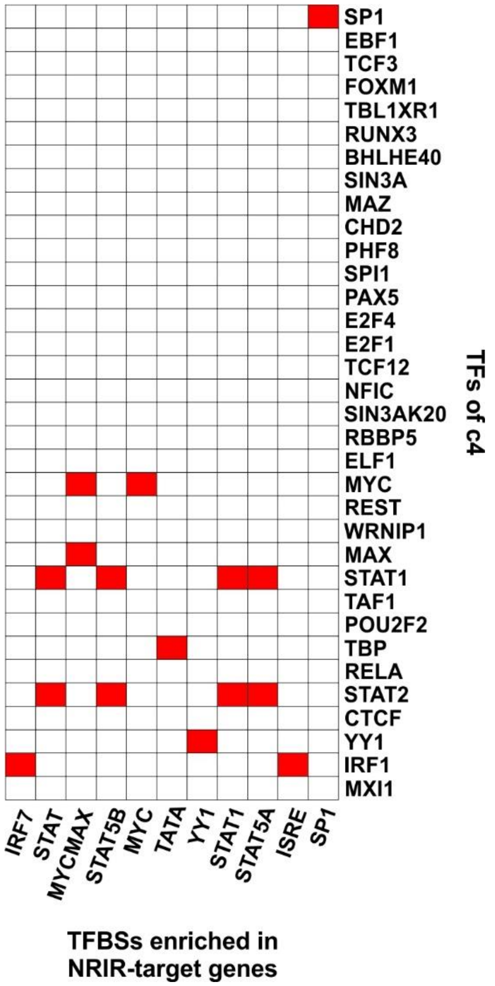

Supplementary Figure S3 Intersection between TFs and TF-bs identified in the promoter of NRIR-modulated ISGs.

TFBs enriched in the promoters of NRIR-target genes (x-axis) and TFs belonging to cluster 4 (Figure 4A) are represented. Red squares highlight TF for which a TFBS was predicted.

Supplementary Table S7 Position of the interacting elements in NRIR sequence

Structural domain<br>Interacting element	Start<br>(nt position)	End<br>(nt position)
**DNA-binding motif**	45	75
**PBE-1**	300	480
**PBE-2**	660	730

nt: nucleotide; PBE: protein binding element

Supplementary Figure S4

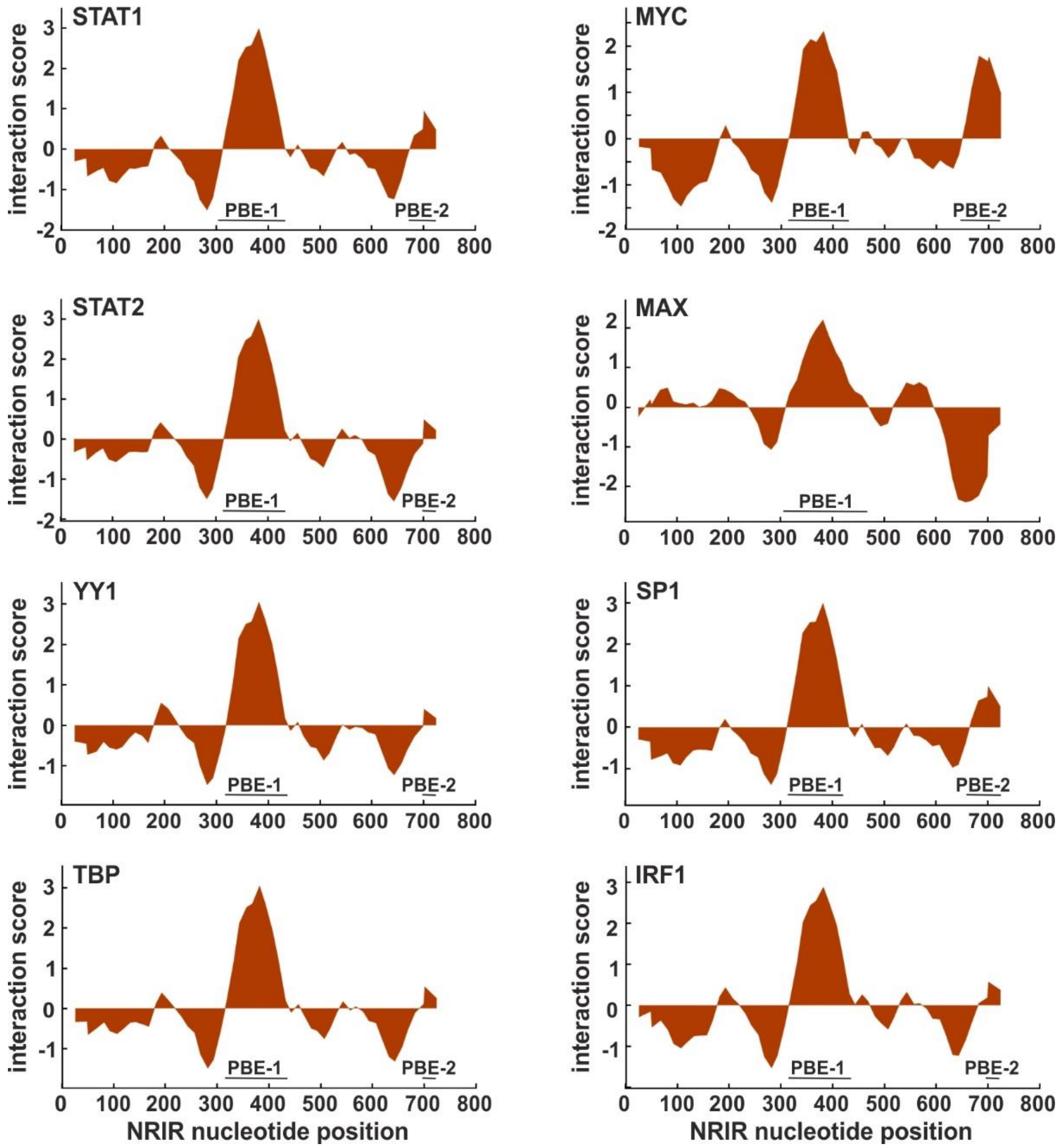

Supplementary Figure4 Interaction profiles of NRIR and selected TFs. Interaction profile of NRIR with STAT1, STAT2, YY1, TBP, MYC, MAX, SP1 and IRF1 was analyzed with catRAPID fragment. Interaction score (y-axis) for each protein with NRIR sequence (x-axis) was plotted. Identified protein binding elements (PBE-1, PBE-2) are highlighted in each graph.

Supplementary references

Castellucci, M., Rossato, M., Calzetti, F., Tamassia, N., Zeminian, S., Cassatella, M.A., et al. (2015). IL-10 disrupts the Brd4-docking sites to inhibit LPS-induced CXCL8 and TNF-alpha expression in monocytes: Implications for chronic obstructive pulmonary disease. *J Allergy Clin Immunol* 136(3)**,** 781-791 e789. doi: 10.1016/j.jaci.2015.04.023.

Mariotti, B., Servaas, N.H., Rossato, M., Tamassia, N., Cassatella, M.A., Cossu, M., et al. (2019). The Long Non-coding RNA NRIR Drives IFN-Response in Monocytes: Implication for Systemic Sclerosis. *Front Immunol* 10**,** 100. doi: 10.3389/fimmu.2019.00100.

Muller, P.Y., Janovjak, H., Miserez, A.R., and Dobbie, Z. (2002). Processing of gene expression data generated by quantitative real-time RT-PCR. *Biotechniques* 32(6)**,** 1372-1374, 1376, 1378-1379.

Rossato, M., Curtale, G., Tamassia, N., Castellucci, M., Mori, L., Gasperini, S., et al. (2012). IL-10-induced microRNA-187 negatively regulates TNF-alpha, IL-6, and IL-12p40 production in TLR4-stimulated monocytes. *Proc Natl Acad Sci U S A* 109(45)**,** E3101-3110. doi: 10.1073/pnas.1209100109.

Zhao, J.C. (2015). nRIP-seq: A Technique to Identify RNA Targets of an RNA Binding Protein on a Genome-Wide Scale. *Regulatory Non-Coding Rnas: Methods and Protocols* 1206**,** 97-106. doi: 10.1007/978-1-4939-1369-5_9.
